# Calcium Phosphate Biomaterials for 3D Bioprinting in Bone Tissue Engineering

**DOI:** 10.3390/biomimetics9020095

**Published:** 2024-02-06

**Authors:** Nelli Tolmacheva, Amitava Bhattacharyya, Insup Noh

**Affiliations:** 1 Convergence Institute of Biomedical Engineering and Biomaterials, Seoul National University of Science and Technology, Seoul 01811, Republic of Korea; 2 Department of Chemical and Biomolecular Engineering, Seoul National University of Science and Technology, Seoul 01811, Republic of Korea; 3 Medical Electronics Research Center, Seoul National University of Science and Technology, Seoul 01811, Republic of Korea

**Keywords:** calcium phosphates, hydroxyapatite, octacalcium phosphate, tissue engineering, bone, 3D bioprinting

## Abstract

Three-dimensional bioprinting is a promising technology for bone tissue engineering. However, most hydrogel bioinks lack the mechanical and post-printing fidelity properties suitable for such hard tissue regeneration. To overcome these weak properties, calcium phosphates can be employed in a bioink to compensate for the lack of certain characteristics. Further, the extracellular matrix of natural bone contains this mineral, resulting in its structural robustness. Thus, calcium phosphates are necessary components of bioink for bone tissue engineering. This review paper examines different recently explored calcium phosphates, as a component of potential bioinks, for the biological, mechanical and structural properties required of 3D bioprinted scaffolds, exploring their distinctive properties that render them favorable biomaterials for bone tissue engineering. The discussion encompasses recent applications and adaptations of 3D-printed scaffolds built with calcium phosphates, delving into the scientific reasons behind the prevalence of certain types of calcium phosphates over others. Additionally, this paper elucidates their interactions with polymer hydrogels for 3D bioprinting applications. Overall, the current status of calcium phosphate/hydrogel bioinks for 3D bioprinting in bone tissue engineering has been investigated.

## 1. Introduction

**Figure 1:**
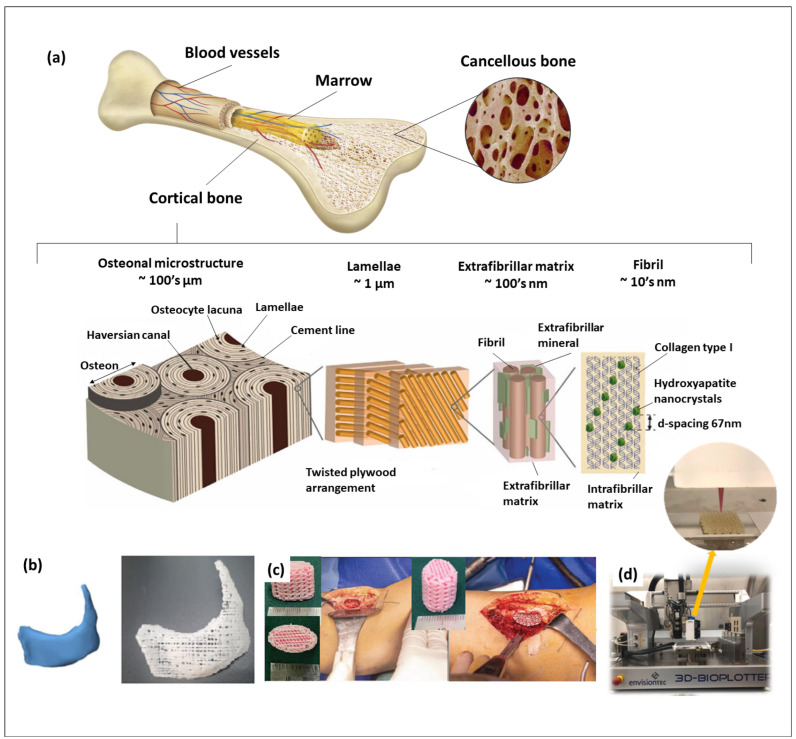
(**a**) Hierarchical bone structure; (**b**) 3D designed patient-specific model and produced PCL/β-TCP scaffold based on this model for treating complex zygomatico-maxillary defects [12]; (**c**) PCL/β-TCP scaffolds with the patient’s autologous platelet-rich plasma [13]; (**d**) 3D-printing process of chitosan/alginate/HAp scaffold for potential cartilage regeneration [14]. (Refs. [12,13,14] were adapted through open access permission).

Utilizing three-dimensional (3D) scaffolds presents significant clinical potential for addressing the biomimetic tissue regeneration of bone defects. Three-dimensional printing techniques have emerged as valuable tools for creating such biomimetic scaffolds with diverse characteristics, such as their mechanical and biological properties. These techniques offer precise control over factors such as overall scaffold geometry, pore size and porosity, as well as physiological and biological properties. Calcium phosphate (CaP) biomaterials are frequently employed in bone tissue engineering as integral components of 3D printing systems for the fabrication of bone tissue scaffolds. When 3D printing technology had just emerged, the focus was on developing CaP-based inks because of their ability to manufacture implants with individual shapes rapidly. When applying 3D printing, it becomes feasible to easily biomimic the mechanical properties and porosity of scaffolds, ensuring their compatibility with patients’ existing bone conditions. This customization helps prevent stress shielding and promotes enhanced bone cell growth and tissue regeneration. Usually, polymers are a necessary part of the inks used in 3D printing. In the case of 3D bioprinting for bone tissue engineering, synthetic polymers have suitable mechanical properties, and bioinertness can be obtained that makes them suitable for implantation. Unfortunately, due to their lack of bioactive properties (in the broad sense of the word), they have some limitations to their use as biomaterials for hard tissue regeneration. However, researchers are now focusing on mimicking real bone structures and properties for future implant resorption and replacing real bone [1]. Bone tissue displays a hierarchical structural organization across various levels, encompassing the macrostructure (cancellous and cortical bone), microstructure (Haversian systems, osteons, single trabeculae), submicrostructure (lamellae) and nanostructure (fibrillar collagen and embedded minerals—HAp nanocrystals as the main element, along with other salts) (Figure 1a). Thus, the CaP-based biomaterials are usually used for cortical bone regeneration, and collagen are employed for cancellous bone regeneration.

The relatively new field of 3D bioprinting is developing rapidly to develop biomimetic tissue-engineering scaffolds. Although CaP-based scaffolds are appropriate for bone tissue engineering, natural bone consists of an inorganic phosphate phase, but its structure is also more complicated, with diverse biomaterials such as collagens, gelatin, fibronectin. Hence, creating 3D-printed scaffolds already loaded with cells can accelerate bone growth and tissue regeneration. But loading inks with cells creates certain restrictions for the bioprinting process and the biomaterials that can be used [2,3,4,5].

Over the last few decades, CaPs have been the preferred biomaterials for creating porous scaffolds to facilitate bone tissue regeneration, given their similarity in chemical structure and biochemical composition to the bone mineral phase [6]. Of the various CaP phases used in 3D bioprinting (Figure 1b–d), the primary focus of research has been on hydroxyapatite (HAp) (Figure 1d) [7], by mimicking its nano/micro-structures (Figure 1a), β-tricalcium phosphate (β-TCP) (Figure 1b,c) [8], a mixture of HAp and β-TCP known as biphasic calcium phosphate (BCP) [9,10,11] and unsintered apatites or calcium-deficient apatites (CDAs). A United States Food and Drug Administration clinical trial search (https://clinicaltrials.gov (accessed on 21 December2023)) indicates numerous investigations of HAp and β-TCP (and their mixture) applied in the healing of bone and teeth diseases such as intervertebral disk degeneration/displacement, mandibular fractures, chronic periodontitis and knee chondral lesions. Most of the clinical trials are at phase 4, at which point the FDA has already approved the drug/medical device material for marketing. During phase 4, additional information about a drug’s/device’s safety, efficacy or optimal use is gathered. Besides this, different combinations of CaPs with polymers or other organic molecules are also studied in phase 2, 3 or 4 and compared with already patented materials (while the phase 2 study gathers preliminary data on whether a drug/medical device works in people who have a certain condition/disease, the phase 3 one is necessary to collect more information about a drug’s/device’s safety and effectiveness by studying different populations and different dosages and by using the drug/device in combination with other drugs/devices). Because of this, with the advancement of 3D bioprinting technologies, the HAp and β-TCP were the first biomaterials used to create three-dimensional scaffolds. However, besides these widely used materials, there are other calcium phosphates which have been investigated for hard tissue-engineering applications; these include calcium pyrophosphate, dicalcium phosphate anhydrous (DCPA), dicalcium phosphate dihydrate (DCPD) and octacalcium phosphate (OCP).

This review paper analyzes recently investigated calcium phosphates for their application as bioink for 3D (bio)printing. Each type of calcium phosphate possesses some exclusive properties that make them preferable biomaterials for bone tissue engineering. The latest applications and modifications of CaP-based 3D-printed scaffolds are also discussed. The scientific background behind the usage of some CaPs compared to others and their interaction with hydrogels are also explained.

## 2. Calcium Phosphates Used in Tissue Engineering

**Figure 2:**
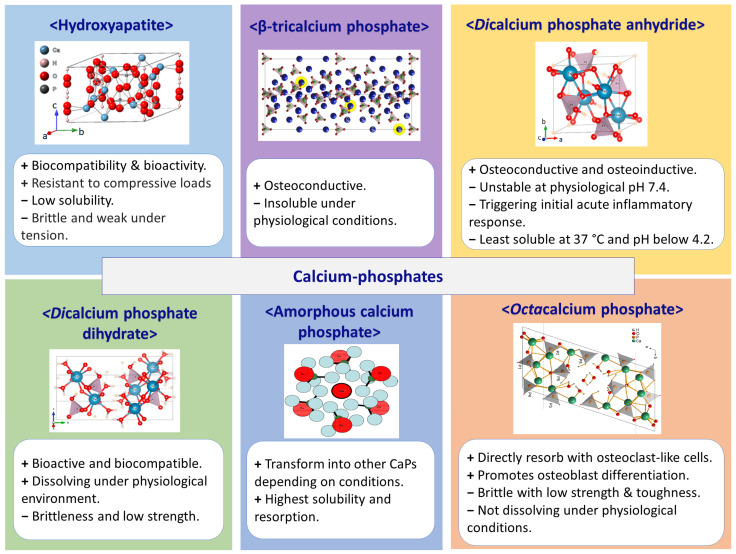
Characteristics of presented calcium phosphates.

Calcium phosphates are extensively employed in 3D printing and bioprinting for skeletal tissue engineering. Due to their properties, some CaPs such as HAp, tricalcium phosphate (TCP) and amorphous calcium phosphate (ACP) are widely used as biomaterials for bone repair. At the same time, other CaPs (dicalcium phosphate anhydrous (DCPA), dicalcium phosphate dihydrate (DCPD) and octacalcium phosphate (OCP)) were discovered earlier but only recently attracted the attention of scientists for applying them in 3D bioprinting. All these calcium phosphates have some distinct properties other than their similar properties (Figure 2). The properties of each calcium phosphate are described in detail in the below subsections.

### 2.1. Hydroxyapatite

Hydroxyapatite (Ca_10_(PO_4_)_6_(OH)_2_) has a Ca/P molar ratio of 1.67, mostly a hexagonal crystal structure and sometimes a monoclinic crystal structure. The primary distinction between these structures lies in the orientations of the hydroxyl groups [15]. HAp is the most significant major bioceramic that can be extensively utilized for biomimicking bone tissue and bone defect repair due to its excellent biocompatibility and bioactivity. In physiological conditions, HAp binds to natural bone by interfering with the surrounding tissues and solution [16]. HAp exhibits an initial mechanical property like cancellous bone—brittle and weak under tension and shear but resistant to compressive loads. The synthetic HAp’s macroporosity, characterized by pores with diameters exceeding 100 μm, facilitates the adhesion, proliferation and differentiation of osteoprogenitor cells. Additionally, it supports revascularization when mixed with other materials and by modifying its structural composition, improving fabrication techniques and growth factor loading and co-cultivating bone regrowth cells to stimulate vascularization [17]. All these lead to the ingrowth of new bone when implanted in vivo. Nevertheless, the crystallinity and high Ca/P ratio reduce the resorption ability of HAp [18]. Due to its stability in an in vivo environment, HAp is frequently utilized as a coating biomaterial on metallic implants to promote regenerative ability in tissue engineering and regenerative medicine [19].

### 2.2. Crystalline Tricalcium Phosphates

In ambient conditions, there are at least two distinct crystalline forms of TCP with a stoichiometric composition of Ca_3_(PO_4_)_2_: α-TCP and β-TCP (β-TCP transforms into α-TCP above 1125 °C). The particle morphology of α-TCP differs significantly from that of CaP crystals synthesized at low temperatures, like HAp, OCP and DCPD crystals. Although α-TCP is metastable at room temperature, its transformation does not occur measurably in dry conditions. Both phases exhibit intermediate solubility compared to orthophosphates, but α-TCP is more reactive in aqueous solutions than β-TCP, readily hydrolyzing into more stable CaPs. The dry synthesis process of α-TCP occurs at high temperatures and, compared to other CaPs, α-TCP cannot be obtained through the precipitation reaction from aqua solution. Due to difficulties in synthesis, α-TCP is a less preferable material for use in tissue engineering. β-TCP crystallizes in the rhombohedral space group and is typically characterized in the trigonal setting. In the past few years, β-TCP has become a highly appealing bone graft substitute, even though β-TCP is insoluble under physiological conditions (pH 7.4) and thus does not align with the in vitro “bioactivity” concept proposed by Kokubo. Although β-TCP does not undergo spontaneous dissolution upon implantation, it is considered osteoconductive as it undergoes resorption through a cell-mediated process with macrophages and multinucleated giant cells (MNGCs). β-TCP’s dissolution ability increases when the surrounding environmental pH decreases. On the β-TCP surface where MNGCs bond, the local pH value becomes 3–4 as cells produce a specific acidic secretion that leads to local dissolution of β-TCP. During this process, β-TCP’s surface is attacked by MNGCs, and the parts that were removed from the surface are absorbed and phagocytosed [20].

### 2.3. Monetite (Dicalcium Phosphate Anhydrous—DCPA) and Brushite (Dicalcium Phosphate Dihydrate—DCPD)

Monetite (CaHPO_4_) belongs to a triclinic crystal system, featuring a crystal lattice composed of a 3D network of tetrahedral phosphate with calcium ions in the interstice. The lack of water molecules results in a more condensed molecular packing arrangement, significantly reducing the unit cell volume, also termed DCPA. In the DCPA structure, three types of hydrogen bonds are present. Because of its structural features, DCPA displays a weak piezoelectric effect and is unstable at physiological pH of 7.4. Among all CaPs, only DCPD is more unstable than DCPA in this physiological condition. However, DCPA turns to the most insoluble CaP at 37 °C and pH below 4.2. Compared to DCPD, DCPA does not easily convert to HAp or other stable CaPs inside the body. DCPA is stable in the local acidic environment because of the rapid release of HPO_4_^2−^ groups. Thus, monetite demonstrates a sustained degradation profile in vivo. During in vitro studies, DCPA showed the ability to promote cell proliferation and increase the expression of osteogenic genes. Implants based on DCPA demonstrated favorable osteoconductive and osteoinductive properties. Monetite grafts, with comparable porosity and structure, exhibit faster in vivo resorption than brushite [21]. The DCPD (Ca_2_H_4_O_6_P^+^—brushite) structure forms dense corrugated sheets composed of parallel chains, where calcium ions are coordinated by six oxygen atoms from the anions and two oxygen atoms from water molecules. Strong interactions between water molecules and adjacent molecules connect the corrugated sheets. In physiological conditions, DCPD is metastable and acidic in nature, making it easily resorbable in the biological system compared to apatite-based CaPs. Due to DCPD’s acidic nature, it can be only obtained in acidic solutions (pH 6 or lower). The mechanical properties of DCPD are less than those of HAp or TCP; DCPD is a brittle and low-strength material [22].

### 2.4. Amorphous Calcium Phosphate

ACP possesses high solubility, attributed to its amorphous structure, the presence of a hydrated layer and structural defects [23]. The absence of periodic long-range orders in ACP facilitates the formation of defects, ultimately increasing solubility and promoting enhanced bioactivity. Choosing ACP as a material for tissue engineering instead of commonly used HAp or β-TCP has the potential to improve bone repair rates through the better biomimicking properties of the scaffolds. However, under specific conditions, metastable ACP transforms into other crystalline calcium phosphates, typically HAp, α-TCP, β-TCP or mixtures thereof [24]. Hence, the high reactivity of ACPs can be leveraged to create diverse bioactive biomaterials. Nowadays, the main application field of ACP is dentistry, and rarely as material for bone grafts due to limits in the formation of 3D structures [25].

### 2.5. Octacalcium Phosphate

OCP (Ca_8_H_2_(PO_4_)_6_∙5H_2_O) is characterized by a repetitive stacked structure comprising an apatite layer resembling a CDA-like structure and a hydrated layer containing a substantial number of water molecules similar to a DCPD-like structure [26]. The two types of layers in OCP alternately stack along the a-axis in the triclinic crystal structure. Due to the similarities in crystallography between OCP and HAp, OCP transforms into HAp epitaxially through HPO_4_^2−^ ion diffusion in the hydrated layer. Consequently, OCP is regarded as a precursor to bone apatite and does not dissolve under physiological conditions. However, OCP is directly resorbed with osteoclast-like cells after implantation. OCP promotes osteoblast differentiation and cell conversion to late osteocytes compared to commercial HAp and β-TCP. Moreover, OCP demonstrates a strong adsorption affinity for a diverse range of proteins, and this affinity is not restricted by serum-derived proteins [27,28,29,30]. Research findings indicate that OCP serves as a superior bone replacement biomaterial compared to other calcium phosphate ceramics. However, OCP is characterized as a brittle material with low strength and poor toughness and, thus, has limitations for clinical applications. However, it does not cause irritation or tissue rejection upon implantation, forms a robust chemical bond with the bone, exhibits a higher likelihood of being resorbed and replaced by newly formed bone and demonstrates enhanced biocompatibility and the ability to induce new bone formation [31].

## 3. Hydrogels Used with Calcium Phosphates in 3D Bioprinting

Hydrogels, derived from synthetic or natural polymers such as collagen, gelatin, fibrin, elastin, agarose, alginate, cellulose and chitosan, are considered optimal bioinks for 3D bioprinting because they mimic natural bone pores and porosity for cell proliferation. These polymers, sourced from both mammalian and non-mammalian origins, fulfill several crucial criteria, including excellent cell adhesion, anticipated biodegradation, non-cytotoxicity, suitable rheological properties (viscous and shear thinning enough for dispensing as a filament), rapid gelation and appropriate strength and stiffness.

Alginate, a polysaccharide extracted from brown algae, serves as a hydrogel in 3D bioprinting and tissue engineering due to its cost-effectiveness, broad compatibility and structural resemblance to proteins existing in the extracellular matrix (ECM). The polymerization of alginate is swift, typically occurring within seconds to minutes upon exposure to divalent cations, enabling diverse ion-doping possibilities. The graft of methacrylate groups allows for the production of hydrogel through light-induced radical polymerization. However, it is worth noting that the inherent structure of alginate lacks binding sites for peptides for cellular interaction, such as Arginine-Glycine-Aspartate (RGD) and YIGSE peptides. Thus, alginate is often conjugated with other cell-adhesive polymers, such as collagen [32], and other functional polymers, such as agarose or hyaluronic acid, with an ECM-based protein or an RGD peptide sequence to facilitate cell adhesion and proliferation. Collagen offers additional advantages, such as low immunogenicity and the promotion of bone regeneration. The use of collagen matrices has demonstrated the ability to influence osteoblast behavior within diverse hydrogels [33]. To achieve rapid gelation, self-healing post-bioprinting and subsequent stabilization through secondary crosslinking, shear-thinning, and self-healing properties, alginate was subjected to methacrylation and oxidation and then ionically crosslinked through an egg-shell model [34]. To make alginate hydrogels more suitable for bone tissue engineering, alginate hydrogels could be incorporated with inorganic particles such as bioglass [35] and kaolin [36] or with organic materials such as cells, drugs and human allograft tissue, as examples [37].

A bacterial hyaluronic acid hydrogel stands out as a promising bioink material for 3D tissue printing. Hyaluronic acid’s presence is crucial for maintaining cartilage homeostasis, promoting chondrogenic phenotypes and stimulating type II collagen production. Because of its low viscosity, challenging gelation process and modest mechanical properties, hyaluronic acid’s application for 3D bioprinting is constrained. To overcome these challenges, hyaluronic acid can be combined with diverse biomaterials, such as peptides and polymeric biomaterials such as chitosan or mechanically stable alginate [38], crosslinked with collagen and poly(ethylene glycol) (PEG) ether tetra-succinimidyl glutarate or with fucoidan through hydrogen bonding [39]. Also, hyaluronic-acid-based hydrogels can be modified with tyramine and then enzymatically crosslinked through the addition of horseradish peroxidase and hydrogen peroxide [40].

Chitosan, a deacetylated form of chitin with the chemical structure of a modified polysaccharide, shares structural similarities with the natural ECM and is a biomaterial for 3D-bioprinting hydrogel. Its biocompatibility, biodegradability, hydrophilicity and non-toxic nature render chitosan an excellent 3D microenvironment for cell encapsulation. Consequently, it is commonly employed as a hydrogel material in tissue engineering and various cell-delivery therapies. Despite these advantages, bioprinted chitosan gel scaffolds suffer from a deficiency in mechanical properties and a lack of osteoconductive properties [41]. Chitosan exhibits solubility in acidic aqueous media at a pH range of 5–6, which is significantly below the optimal pH for cell culture. It can undergo a sol–gel transition through various gelation techniques, including ionotropic gelation, polyelectrolyte complexation and self-assembly. However, these methods rely on the ability of polyelectrolytes to crosslink in the presence of counterions. Consequently, other polymers with negatively charged ions that electrostatically interact with the –NH_2_ units of chitosan chains, i.e., the deacetylated group of chitin side chains, are required for gelation. For instance, introducing glycol groups to the chitosan polymer chain can render chitosan water soluble. Furthermore, the methacrylation of glycol chitosan provides the opportunity for crosslinking hydrogels under ultraviolet or visible light [42]. Chitosan/gelatin nanofibrouscan be formed through the electrostatic interactions between a positively charged (chitosan) and a negatively charged (alginate) polymer. By incorporating these nanofibrous structures, thermosensitive chitosan hydrogels can be developed. Furthermore, the addition of nanohydroxyapatite can enhance the mechanical properties of chitosan-based hydrogels [43].

Gelatin hydrogels find widespread use in tissue-engineering applications owing to their excellent cell-adhesive scaffold compatibility, support for proliferation and cost-effectiveness, despite limitations in mechanical properties. Gelatin dissolves in warm water and transforms into a hydrogel upon cooling to room temperature due to the formation of a weak hydrogen bond network. However, when gelatin is combined with alginate, silk fibroin, chitosan or other natural polymers, the interactions, including covalent, hydrogen and van der Waals forces, are enhanced. This results in hydrogels with superior mechanical, functional and biocompatible properties compared to pure gelatin hydrogels. Also, gelatin can be combined with synthetic polymers to improve the ink’s properties, because usually synthetic polymers lack bioactive properties but have high mechanical characteristics [44]. The gelatin-based hydrogel can be crosslinked with a combination of tannic acid and ferrous sulfate to achieve better extrudable and stable properties for 3D bioprinting [45]. Gelatin alginate hydrogel stands out as one of the most prevalent and user-friendly bioinks. Its printing temperature (37 °C) renders it compatible with living cells and heat-sensitive substances, including drugs. While it is solid at room temperature, the solidification process can be accelerated by reducing the temperature of the collector [46]. A combination of gelatin-based hydrogel with gelatin methacryloyl can undergo photo-crosslinking in the presence of a water-soluble photo-initiator under blue-light irradiation [47]. By controlling a complex polymer system, optimal properties can be achieved. A composite bioink with hydrogel, composed of gelatin, carboxymethyl cellulose and alginate, serves as biofiller in a customized human knee meniscus negative mold derived through an indirect rapid prototyping process and as a bioink in the 3D-printing process [48]. Crosslinked with ginipin gelatin hyaluronic acid, a bioresponsive hydrogel demonstrated enhanced stability and extrudable properties in 3D bioprinting [49]. A gelatin methacrylate-hyaluronic acid methacrylate-chondroitin sulfate methacrylate hydrogel system loaded with modified proteins and polysaccharides exhibits structural integrity, mechanical stability and long-term cell viability [50].

## 4. Status of Calcium Phosphate Biomaterials in 3D (Bio)Printing Applications

### 4.1. Hydroxyapatite (HAp)-Based 3D-Printed Scaffolds

Currently, 3D-bioprinting technology is extensively researched in the medical field for manufacturing scaffolds. This is primarily due to its excellent controllability regarding scaffold design, allowing for mimicking the defect’s shape and reaching the necessary scaffold’s inner structure. This precision enables the stable and accurate placement of tissue-engineering scaffolds in defects, facilitating high bone conduction and tissue regeneration. Correspondingly, HAp, as the biomaterial with the closest chemical structure to natural bone, was one of the first calcium phosphates that was applied to the 3D-printing process. However, the application of pristine HAp in clinical practice is limited due to its intrinsically poor mechanical properties, including low fracture toughness and tensile strength. Thus, many improvement approaches have been implemented. In Table 1, some of the recent significant studies of HAp-containing 3D-printed scaffolds are represented.

Creating a HAp-based composition of the scaffolds with polymers improves the mechanical properties and enables the production of multilayer compositions for 3D printing [14,54,55,56,58]. To increase the mechanical properties of 3D-printed HAp/polymeric biomaterials, in some research, graphene oxide was added [57]. Replacing Ca ions in the HAp frame structure with other metal ions is commonly employed to add crucial ions that can greatly affect material properties. Cerium-doped HAp/gelatin methacryloyl (GelMA) 3D scaffolds have exceptional structural integrity and homogeneity, which is a great point of compatibility for the 3D-printing process. In addition, substitution with cerium ions enhanced the cell viability and proliferation potency of Ce-HAp/GelMA composites while maintaining a low cytotoxic potential [52]. Magnesium and zinc have been shown to have positive effects on the osteogenic differentiation process through biological studies of gene expression [53]. Both stoichiometric and Sr-containing HAp crystals allow particle-dependent enhancement of the scaffolds’ printing quality. Also, by substituting a particular amount of Sr in HAp, the scaffolds’ swelling behavior and ion release become more regulated [60]. For promoting bone formation, HAp/polymer 3D scaffolds were loaded with drugs, which were released after implantation. Drug release behavior can be regulated by coordinating the scaffold’s 3D structure, pore size and shape [51,59].

### 4.2. Beta-Tricalcium Phosphate (β-TCP)-Based 3D-Printed Scaffolds

The lack of HAp degradation can lead to issues in tissue engineering such as a lack of tissue regeneration, bone deformities and an increased long-term risk of bone fractures around HAp-based bone substitutes. It might also necessitate the permanent use of osteosynthesis fixation, even when it is initially intended for temporary use. In contrast to sintered HAp, β-TCP is resorbable and readily replaced by new bone. As well as hydroxyapatite, tricalcium phosphate is used with printable polymers to improve the lack in the polymers’ mechanical properties and enhance the bioactive properties of 3D-printed scaffolds. Below, there are recent works related to 3D-printed scaffolds containing β-TCP, and their short descriptions are collected in Table 2.

Thus, Ye et al. incorporated poly(hydroxyl alkanoate) with β-TCP. Obtained scaffolds displayed compressive strength like that of natural bone [61]. Thermoplastic polyurethane was blended with poly(lactic-co-glycolic acid) (PLGA)/β-TCP, and manufactured 3D-printed scaffolds not only demonstrated superior mechanical properties compared to a commercially available calcium phosphate ink (OsteoInk^®^) but also showed satisfactory cytocompatibility and osteoconductive properties [62]. Polycaprolactone (PCL)/β-TCP scaffolds have garnered considerable attention in tissue engineering due to their ability to stimulate the formation of a mineralized matrix and support osteogenic cell differentiation. Scaffolds produced from poly(tri-methyl carbonate) (PTMC)/poly(ε-caprolactone)/β-TCP demonstrated low cytotoxicity and exceptional biocompatibility. Moreover, these scaffolds facilitated the proliferation of osteoblast cell lines MC3T3-E1 and rBMSC (stem cells), exhibiting improved cell adhesion, penetration and overall proliferation [63]. However, during the 3D-printing process, PCL tends to cover the bioceramic, which leads to a diminished interaction between the bioceramic and cells. To solve this problem, Liu, K. et al. introduced PEG into 3D-printed PCL/β-TCP scaffolds. The integration of PEG-coated β-TCP structures enabled the controlled release of β-TCP from within the scaffolds, effectively overcoming the issue of inadequate β-TCP release when solely covered by PCL. The resultant PCL/β-TCP/PEG scaffolds exhibited excellent wettability, a slight reduction in mechanical properties and enhanced in vitro mineralization. Cell viability assessments demonstrated significant cell proliferation after 5 days, attributed to the incorporation of PEG and β-TCP materials in the PCL scaffolds [64]. Some 3D-printed scaffolds have already been tested in clinical settings; for example, Jeong, W.S. et al. evaluated the efficacy of a 3D-printed, patient-specific PCL/β-TCP scaffold for treating complex zygomatic–maxillary defects. Eight patients who underwent immediate or delayed maxillary reconstruction using personalized PCL/β-TCP 3D-printed implants between December 2019 and June 2021 took part in the research. The reconstruction procedures utilized various techniques, such as bone grafts, fasciocutaneous free flaps and fat grafts. Volume analysis revealed satisfactory conformity between preoperative simulation and actual implant volume, with a mean volume conformity of 79.71%, ranging from 70.89% to 86.31% [12]. To enhance the scaffold’s mechanical properties, not only can HAp or β-TCP be added but carbon nanotubes are often used as grafting materials. In a study, PCL was mixed with carbon nanotubes (CNTs) and calcium phosphates (HAp and β-TCP) [65]. Manufactured 3D-printed scaffolds showed early in vivo osteogenic and inflammatory responses. The increased scaffold endurance, possibly due to the higher stiffness imparted by CNTs, facilitated the efficient absorption of ceramic materials. Both scaffolds showed elevated expressions of genes related to osteogenesis, tissue formation and mineralization. Sometimes the addition of β-TCP can lead to deterioration of the mechanical properties of the polymer. Polylactic-acid-based 3D-printed scaffolds with β-TCP exhibited lower mechanical properties compared to pure polylactic acid scaffolds [66]. Along with CNTs, lignin can be incorporated to enhance mechanical properties. The incorporation of lignin enhanced the mechanical strength of the sodium acetate/β-TCP 3D-printed scaffolds and created an environment more conducive to the adhesion and proliferation of cells [67]. Incorporating 3D-printed scaffolds with drugs is another method to increase bone formation capability. Xu and team incorporated the adenosine receptor indirect agonist dipyridamole into a poly(vinyl alcohol)/β-TCP composite biomaterial using 3D-printing technology. The findings revealed that the inclusion of the drug enhanced the hydrophilicity of the scaffold, promoted cell proliferation and adhesion and notably stimulated the osteogenic differentiation of stem cells [68].

**Table 2:** Recently investigated 3D-printed scaffolds incorporating β-tricalcium phosphate.

Polymer	Filler Used	β-TCP Concentration/Particle Size	Highlights	Scaffold’s Pore Size (Porosity, %)	Ref.
Poly(hydroxyalkanoates) (PHAs)		0 wt%, 5 wt%, 10 wt%, 20 wt% and 30 wt% of PHA/10–20 μm	The addition of β-TCP significantly improved the proliferation, adhesion and migration of MC3T3-E1 cells. The obtained scaffolds presented compressive strength compatible with natural bone.	~400 μm	[61]
Poly(lactic acid) (PLA)		15 vol% β-TCP/(d50)—5 (±2) µm	Higher nozzle temperatures helped enhance the tensile strength of the printed parts. TCP–PLA printed samples experienced lower mechanical properties than PLA.		[66]
Polycaprolactone		20% wt%	The PCL/β-TCP scaffold can provide durable support and enhance bone formation in complex zygomatic–maxillary defects.	500 µm	[12]
Poly(tri-methyl carbonate) (PTMC)/Poly(ε-caprolactone) (PCL)		0–25% wt%	Provides good porous growth microenvironments and mechanical support for MC3T3-E1 cells and rBMSCs and enhances the proliferation of osteoblast cells.	PCL/25%TCP: (66.43 ± 2.56%) PTMC/25%TCP: (58.9 ± 2.81%) PTMC/PCL/25%TCP (48.0 ± 1.84%)	[63]
Polycaprolactone (PCL)	Poly(ethylene glycol)	10 wt%/9.3 μm	Compared with the PCL scaffolds, the PCL/TCP/PEG scaffolds exhibited good wettability (contact angle decreased from 85 °C to 0 °C, showing complete wettability). The alkaline phosphatase content of the PCL/TCP/PEG scaffold increased by 2.5 times after 14 days of co-culture compared with the PCL scaffold.	550 μm	[64]
Polycaprolactone (PCL)	Carbon nanotubes (CNTs)/HAp	10 and 20 wt%	Enhances gene expression.	350 µm	[65]
Poly(lactic-co-glycol acid) (PLGA)	Elastic thermoplastic polyurethane	20% *w*/*v*	Better mechanical properties compared to OsteoInk^®^.	Printing porosity: 100 nm–1 mm Surface porosity: 2–50 mm	[62]
Sodium acetate (SA)	Lignin	70–80%	LG-containing scaffolds show a 15% increase in mechanical strength. The capacity to promote the osteoblasts’ adhesion and proliferation as well as their bioactivity (formation of hydroxyapatite crystals) was improved.	(37–41%)	[67]
Polyvinyl alcohol (PVA)	Dipyridamole	<100 μm	Improving the scaffold’s hydrophilicity and promoting cell proliferation and adhesion and significantly inducing osteogenic differentiation of stem cells were fixed.	~500 μm	[68]

### 4.3. Biphasic Calcium Phosphate (BCP)-Based 3D-Printed Scaffolds

BCP, as well as HAp and β-TCP, are widely used biomaterials for bone repair in clinical applications. BCPs typically refer to mixtures of HAp and β-TCP with varying ratios. The advantage of BCP lies in combining the stable mechanical properties and excellent bioactivity of HAp with the rapid degradation property of β-TCP. Numerous studies have demonstrated that BCP exhibits superior osteoinductive properties compared to pure HAp or β-TCP alone. Doping BCP with ions can enhance its biological and mechanical properties, making it a more effective material for bone repair. BCP scaffolds doped with Zn (2.5 mol%) show high osteoinductive activity, and the formation of new bone occurs more rapidly [69]. The incorporation of magnesium proved advantageous for the sintering process of BCP, leading to improved scaffold density and a reduction in the initial degradation rate. However, the introduction of Mg disrupted the stability of the crystal structure, which led to the degradation of BCP scaffolds in the later stages. Furthermore, the addition of magnesium through doping enhanced the vascular activity of endothelial cells in vitro [70]. The 3D-printed BCP scaffolds exhibit significant potential for clinical applications in bone tissue engineering. However, achieving vascularization in the scaffold remains a critical step for effective bone regeneration, and its control still poses a challenging problem for researchers. An effort was made to enhance the vascularization of scaffolds by loading a 3D-printed BCP scaffold with platelet lysate(PL)/gelatin methacrylate (PL/GelMA) [71]. Comparatively, the mechanical properties of the PL/GelMA/BCP scaffold were slightly improved when compared to pure BCP scaffolds. However, the PL/GelMA/BCP scaffold demonstrated increased favorability for cell adhesion and growth. Upon co-culturing with the PL/GelMA/BCP scaffold, the proliferation and expression of angiogenesis-related genes in HUVECs were enhanced. Additionally, the PL/GelMA/BCP scaffold exhibited favorable slow-release behavior of factors, and the scaffold extract promoted the formation of tube structures in HUVECs. In another study, the introduction of graphene oxide (GO) into the 3D-printed BCP scaffolds showed good biological effects, such as stimulating the differentiation of rat bone marrow stem cells (BMSCs) and promoting the migration of HUVECs for enhanced bone repair [72]. Also, 3D-printed scaffolds with mixtures of natural polymers (chitosan and silk fibroin) enhance the metabolic activity of human osteoblasts cultivated on the scaffolds with varying macropore sizes. This improvement aligns with the complete cellular coverage observed on the scaffold surfaces [73]. In a study, stromal vascular fraction heterogeneous cells obtained from autologous adipose tissue were incorporated into hyaluronic acid/gelatin/BCP (HyA-Gel/BCP) scaffolds for bone regeneration [74]. Upon in vivo implantation, the autologous stromal-vascular-fraction-loaded scaffolds demonstrated remarkable bone regeneration compared to the scaffolds without stromal vascular fraction loading.

### 4.4. Octacalcium Phosphate (OCP) as Biomaterial

**Figure 3:**
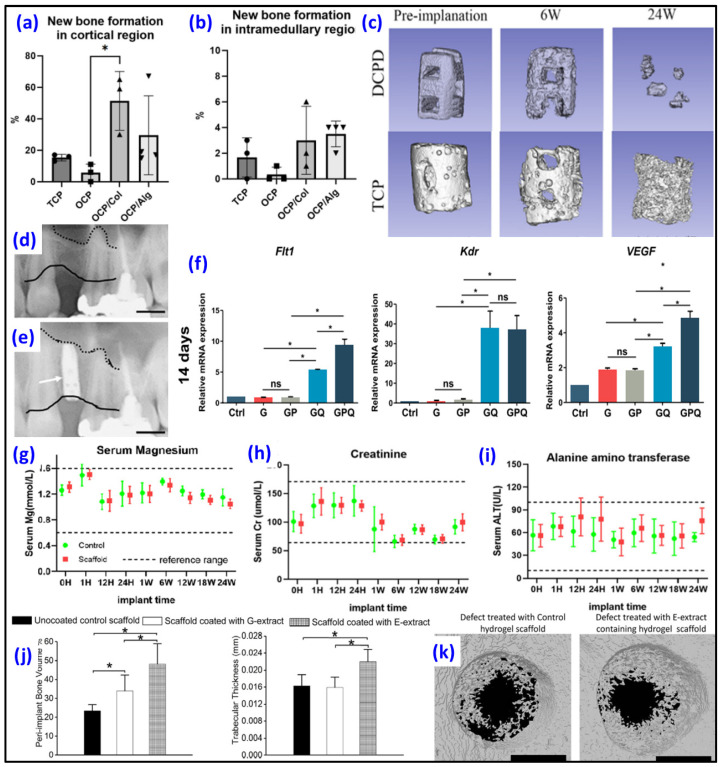
Histo-morphometric analysis of newly formed bone in the cortical (**a**) and intramedullary (**b**) regions filled with β-TCP, C-OCP, OCP/Col and OCP/Alg composite scaffolds (* *p* < 0.05) [77]; (**c**) Degradation of TCP scaffold and 3D-printed Mg scaffolds covered with DCPD in vivo after implantation (6 and 24 weeks) (Reprinted/adapted with permission from Ref. [83]. Copyright 2023, Elsevier); (**d**) X-ray of the patient before OCP/Col implantation and (**e**) at 6 months after OCP/Col implantation with dental implant (black lines—alveolar crest, dotted lines—sinus floor, white arrow—dental implant) [78]; (**f**) Results of expression of angiogenesis-related genes that were cultured with VEGF-mimetic peptide-loaded OCP/gelatin hydrogels after 14 days (* *p* < 0.05, ns—no significant difference) [81]; (**g**) Serum Mg ion concentration after in vivo implantation of Mg-based scaffold coated with DCPD in vivo at different time points (control group—green, with scaffold group—red), creatinine (Kidney function indicator) (**h**) and alanine amino transferase (Liver function indicator); (**i**) analysis of 3D-printed Mg scaffolds covered with DCPD after surgery (Reprinted/adapted with permission from Ref. [83]. Copyright 2023, Elsevier); (**j**) In vivo regenerative performance of DCPD/Pluronic-acid-based hydrogel scaffolds coated with bone ECM extracts and uncoated (* *p* < 0.05) and (**k**) Micro-CT reconstructions of tibial cortical defects filled with control DCPD/Pluronic-acid-based hydrogel scaffolds (without extracts) and scaffolds containing E-extract (Reprinted/adapted with permission from Ref. [84]. Copyright 2023, Elsevier). (Refs. [77,78,81] were adapted through open access permission).

Like in other calcium phosphates, calcium ions in the OCP structural lattice can be substituted with different ions. Cell viability significantly increased in the Sr-substituted OCP compared to the undoped OCP, indicating the absence of an impact from OCP-Sr on the content of acidic compartments, as well as the potential absence of or even an inhibitory effect on the production of reactive oxygen species [75]. Xu et al. investigated 3D-printed PLA/Lanthanum (La)-doped OCP scaffolds. The La-OCP/PLA scaffolds demonstrated noticeable mineralization effects and sustained release of La^3+^ on cell behaviors. These scaffolds exhibited a uniform structure with a rough micro-surface topography, providing a suitable pathway for the adhesion, growth and proliferation of BMSCs [76]. However, OCP’s practical use is limited due to handling difficulties. Three types of OCPs—cemented OCP (C-OCP), C-OCP with collagen (OCP/Col) and synthetic OCP (S-OCP) with alginate (OCP/Alg)—were prepared and compared with commercially available β-TCP to assess their potential in accelerating bone formation in defective rat tibias [77]. Among the samples, the OCP/Col composite exhibited significant bone formation compared to those of the β-TCP or OCP/Alg samples (Figure 3a,b). However, its fast degradation makes it unsuitable for use in conditions involving mechanical stress in clinical orthopedic settings. The OCP/Alg samples, on the other hand, demonstrated efficacy comparable to the β-TCP samples in accelerating bone formation (Figure 3b) while exhibiting better biodegradability suitable for clinical applications in various circumstances. In another study, Kawai et al. reported the clinical application of the OCP/collagen (OCP/Col) composite for bone augmentation in major oral and maxillofacial surgeries. The OCP/Col sample was employed in a clinical trial for bone augmentation, specifically in sinus floor elevation (one- and two-stage procedures), socket preservation, cyst and alveolar cleft cases. Before implantation of OCP/Col, the distance from the alveolar crest to the sinus floor was 6 mm (Figure 3d) and became 10 mm 6 months after surgery for a sinus floor elevation (Figure 3e). While one-stage sinus floor elevation, cyst and alveolar cleft cases achieved success according to the predefined criteria, the two-stage and socket preservation groups initially did not meet the criteria. However, further assessment revealed the effectiveness of OCP/Col in these groups, ultimately meeting the success criteria [78]. To enable the local release of therapeutic concentrations into the bone defect after surgery, in a study by Kuvshinova et al., OCP bone grafts were loaded with the cytostatic drug cisplatin [79]. The study found that zoledronic acid altered the physicochemical and bioactive properties of the OCP ceramics and extended the release of cisplatin from the ceramics. In vitro and in vivo experiments confirmed the biocompatibility, osteoconductivity and osteoinductivity, along with the cytostatic and antitumor properties, of the resulting biomaterials. The irregular nature of bone defects presents a significant challenge in bone defect repair. Addressing this challenge, chitosan/polydopamine/OCP microcarriers, which were designed to closely resemble bone composition while adapting to various bone defect shapes and sizes, demonstrated excellent biocompatibility, fostering favorable conditions for cell adhesion and proliferation. Furthermore, assessments through alkaline phosphatase (ALP) and Alizarin Red S staining indicated that the inclusion of OCP significantly boosted the osteogenic differentiation of BMSCs [80]. In another study, hydrogel loaded with a VEGF-mimetic peptide (QK) demonstrated the ability to enhance tube formation in human umbilical vein endothelial cells (HUVECs), elevate the expression of angiogenesis-related genes (Flt1, Kdr and VEGF) in BMSCs (Figure 3f) and recruit BMSCs, collectively contributing to the establishment of an osteogenic microenvironment for bone repair [81]. Bordbar-Khiabani et al. explored 3D-printed titanium alloys that were covered with alginate hydrogels with and without OCP to improve their resistance to corrosion under simulated normal, inflammatory and severe inflammatory conditions in vitro [82]. The findings indicated that alginate hydrogel/OCP coatings exhibited higher hydrophilicity compared to that of pure alginate hydrogel. Further, the crystallinity of the OCP phase increased when exposed to simulated biological media. While corrosion resistance decreased in inflammatory environments, the hydrogel coatings on the 3D-printed Ti layers shifted the corrosion potential toward more noble values, resulting in reduced corrosion current density across all simulated solutions. Notably, the presence of OCP particles in the alginate hydrogel matrix increased electrical charge transfer resistance at the interface between the substrate and coating more than hydrogel alone.

### 4.5. Dicalcium Phosphate Dihydrate (DCPD) as Biomaterial

The results from electrochemical corrosion measurements of Mg plates and in vitro immersion experiments demonstrated that coatings of DCPD not only effectively controlled the corrosion of biodegradable Mg but also displayed self-healing capabilities in both Hanks’ and normal saline solutions. The presence of local alkaline conditions resulting from scratches facilitated the formation of anticorrosive product layers due to the supplementation of calcium and phosphorus from DCPD. This led to the sealing of scratches, achieving autonomous self-healing without the need for additional corrosion inhibitors in DCPD [85]. In another study, a 3D-printed scaffold covered with DCPD achieved 5.38 MPa strength, and the degradation rate decreased after coating [83]. The implantation of the scaffold did not lead to an elevation in the Mg ion concentration (Figure 3g), and no toxic damage was observed in the kidney (Figure 3h) or liver (Figure 3i). TCP scaffold and Mg 3D-printed scaffolds covered with DCPD showed a close degradation rate 6 weeks after surgery, but at 12 weeks and 24 weeks, scaffolds covered with DCPD exhibited more rapid resorption compared to TCP scaffold (Figure 3c). DCPD and calcium silicate were used as components for the incorporation of sodium D-mannuronate and L-guluronate/gelatin hydrogels [86]. The findings from cell mortality assessments validate that the release of biologically active ions from mineral-filled hydrogels, particularly those with the highest percentage of CaSi-DCPD, results in decreased cell mortality and enhanced biological properties of the materials, as indicated by the increased gene expression. Mansour et al. investigated the ability to modify the calcium phosphate surface to mimic the bone ECM through the incorporation of DCPD/Pluronic-acid-based hydrogel with bone ECM proteins: collagenous proteins (G-extract) and non-collagenous proteins (E-extract). Obtained scaffolds with bone extracts, particularly those enriched with calcium-binding proteins, significantly influenced their interaction with plasma proteins in vitro, particularly those associated with the innate immune response. In vivo, a dampened inflammatory response to the bioceramic scaffolds and an increased formation of new bone around the scaffolds (Figure 3j), supported by heightened osteoblastogenesis and reduced osteoclastogenesis, were registered. In particular, 3D-printed scaffolds with E-extract exhibited significant cortical bone regeneration in a rat tibial defect in vivo experiment compared to G-extract-containing scaffolds or those without extract (Figure 3k) [84].

## 5. CaPs Incorporated Hydrogels for 3D Bioprinting

The potential applications of 3D-bioprinted nanocomposite hydrogels are extremely wide. The appropriate combination of CaP and hydrogel-based 3D bioprinted scaffolds can mimic different real bone structures. CaP nanoparticles not only enhance the physical and chemical characteristics of hydrogel materials but also augment cellular activities. Significant recent investigations about the utilization of CaP in conjunction with hydrogels for 3D bioprinting are examined and summarized in Table 3.

**Figure 4:**
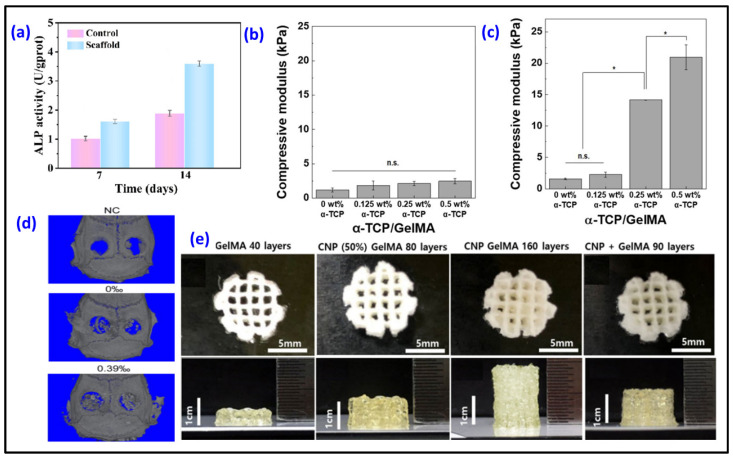
(**a**) The ALP activity after BMSCs were cultured on HAp/collagen scaffolds for 7 and 14 days [87]; (**b**) The compressive modulus of α-TCP/GelMA hydrogel samples containing 0, 0.125, 0.25 or 0.5 wt%, α-TCP before the calcium phosphate phase transition (0 h) (n.s. = not significant) and (**c**) after the calcium phosphate phase transition (36 h) (* *p* < 0.05) [95]; (**d**) Micro-CT images of implanted composite hydrogel scaffolds in a rat defect model after 30 days [88]; (**e**) The heights of the 3D-printed samples that were produced from GelMA and GelMA containing calcium phosphate micro/nanoparticles [96]. (Refs. [87,88,95,96] were adapted through open access permission).

Collagen, a widely used scaffold in tissue engineering, faces limitations in terms of printability and mechanical strength, hindering its effective application in 3D bioprinting. Researchers printed HAp/collagen scaffolds into a gelatin support bath to enhance collagen mechanical properties [87]. Utilizing a gelatin support bath proved effective in supporting the 3D printing of composite patterns, ensuring high fidelity and enabling the printing of cell-laden scaffolds at room temperature. The incorporation of nano-HAp with collagen imparted favorable mechanical properties to the biomimetic bone 3D scaffold. Also, in vitro cell assessment revealed excellent cytocompatibility and increased ALP expression (Figure 4a) in the HAp/collagen scaffold. In a study, 3D-printed scaffolds with nanohydroxyapatite (nHAp)/sodium alginate/gelatin hydrogel were loaded with emodin [88]. Emodin has demonstrated a direct and effective promotion of osteoblast proliferation, migration, differentiation and mineralization. Moreover, the emodin-containing scaffolds demonstrated effective modulation of macrophages. Expression of M1-related genes (IL-6, iNOS and TNF-α) notably decreased, and the expression of M2-related genes (ARG-1, CD206) increased, thereby indirectly fostering osteogenesis. After 30 days of implantation, 0.39% of emodin-loaded scaffolds showed better bone regeneration compared to that of the non-loaded scaffolds and control sample without any scaffolds (Figure 4d). Decellularized extracellular matrix (dECM), gelatin, quaternized chitosan and nHAp-based 3D hybrid scaffolds loaded with exosomes display an interconnected pore structure and suitable degradability, with over 61% of the scaffold dissolved after 8 weeks [89]. Additionally, the hybrid scaffold (containing 20% nHAp) exhibited effective antibacterial properties, excellent hemocompatibility and biocompatibility. Koo et al. investigated the conditions of 3D bioprinting to create structures laden with cells that resemble meringue, promoting enhanced osteogenic activity [90]. Ladened with MG63 cells and human adipose stem cells, 3D-printed collagen/HAp scaffolds demonstrated exceptional metabolic and osteogenic activities, attributed to the synergistic effects of efficient cell-to-cell interactions and the stimulation provided by HAp released from the porous structure. To achieve homogeneous and delicate mixing of bioinks containing nano/microparticles and cells, A. Bhattacharyya and colleagues developed a semi-automated twin screw extruder head and printed alginate/α-TCP/osteoblast cell scaffolds with a manufactured system [91].

In another study, Ghahri et al. developed the process of making a bioprinted scaffold that mimics the structural unit of compact bone, osteon. To achieve this, a special quadruple-layer core–shell nozzle was utilized. A scaffold–cell construct was produced using PEG as a hollowing agent in the first layer. The second layer (vasculogenic layer) consisted of a gelatin methacryloyl (GelMA)/alginate blend hydrogel encapsulating HUVECs with vascular endothelial growth factor (VEGF) nanoparticles to mimic vascular vessels. The outer osteogenic layer comprised GelMA/alginate blended hydrogel containing hMSCs to imitate lamella. Two types of bone minerals, whitlockite and HAp, were included in the osteogenic layer to induce osteoblastic differentiation and enhance mechanical properties. The bioinks, incorporating two types of encapsulated cells, exhibited suitable mechanical strength to create an optimal niche for the growth and differentiation of each cell. They demonstrated stable swelling behavior, an appropriate degradation rate and shear-thinning behavior, essential for supporting encapsulated cells and the biofabrication process. The significant increase in vascular endothelial growth gene expression could signal upregulate cellular activity associated with vascularization in the scaffold–cell construct [92]. GelMA/gelatin/HAp 3D-printed scaffolds with loading of MC3T3-E1 Subclone 4 mouse calvarial osteoblasts were produced [93]. The incorporation of HAp into GelMA/gelatin hydrogels led to several improvements: it reduced hydrogel swelling, enhanced resistance to enzymatic degradation, promoted osteoblastic differentiation and mineralization and increased the expression of osteogenic genes. Importantly, these enhancements were achieved while maintaining equivalent cell viability and proliferation compared to hydrogels without HAp. Vurat and colleagues aimed to create a 3D-bioprinted microtissue model representing the periodontal ligament alveolar bone biointerface. The periodontal ligament layer was generated by bioprinting GelMA bioink with human periodontal ligament fibroblasts (hPDLFs), while the alveolar bone layer was formed by bioprinting GelMA/HApMNP composite bioink with both human osteoblasts (hOBs) and hydroxyapatite–magnetic iron oxide nanoparticles (HApMNPs). The obtained hybrid constructs exhibited higher compressive strength compared to that of the GelMA constructs. In addition, the microtissue construct with two layers exhibits permeability to cell migration [94]. To enhance the bioprintability of GelMA–hydrogel bioinks, the transition from one form to another under environmental conditions was exploited by incorporating CaP [95]. MC3T3-E1 was added to the GelMA hydrogel containing α-TCP (<0.5 wt%). Calcium-deficient HAp was obtained through the α-TCP phase transition in the cell culture medium for 36 h. The compressive modulus of 0.5 wt% α-TCP/GelMA scaffolds increased approximately 18 times compared with pure GelMA (Figure 4b,c). While cell proliferation experienced a decrease in the initial stages of cultivation, there was a significant increase in both osteogenic differentiation and mineralization activities following the CaP phase transition. The inclusion of α-TCP not only enhanced the printability and fidelity of GelMA but also improved the structural stability and compressive modulus, reaching approximately six times higher values after three weeks of culturing. In a study, GelMA-based bioinks were incorporated with amorphous calcium phosphate micro/nanoparticles (CNPs) to reinforce mechanical properties [96]. To reduce the undesired impact of CNPs on cell culture during bioink preparation, CNPs were synthesized using a one-pot process that included positioning of calcium ions in the gelatin chemical structure and growth of CNPs through phosphate addition, with gelatin serving as a stabilizing agent in the matrix. Thus, the size distributions and shapes of the nanoparticles can be controlled based on the concentrations of gelatin. The obtained 3D-printed scaffold exhibits significantly improved mechanical and biological properties, as well as enhanced printability, compared to the sample that was obtained through the simultaneous process of both mixing separate nanoparticles and GelMA and photo-crosslinking. While a scaffold with 40 layers could be produced from pure GelMA ink, the crosslinked GelMA–hydrogel mixed with CNP scaffold could be printed with up to 90 layers. The samples fabricated from the inks that were prepared through a one-pot process with CNP+GelMA and CNP-dispersed GelMA scaffolds can achieve 80 and 160 layers, respectively (Figure 4e). Further study showed that CNP-GelMa and CNP+GelMa groups exhibited great bone regeneration after implantation for 2 weeks in rabbit calvarial defects [98].

Hao and colleagues reported successful in vivo implantation of 3D-printed PCL/β-TCP scaffolds loaded with the patient’s autologous platelet-rich plasma (PRP) to regenerate bone defects that occurred as the result of tibial tumor resection [13]. Kim et al. investigated the difference in the properties of scaffolds loaded with hASCs and HUVECs and scaffolds loaded only with hASCs. Implanted hASC/HUVEC-laden scaffolds demonstrated significantly greater levels of new bone formation and angiogenesis in comparison to the constructs containing only hASCs [97].

## 6. Discussion and Perspective

### 6.1. Issues of CaP/Hydrogels for 3D Bioprinting

In the realm of 3D-bioprinting materials, hydroxyapatite and tricalcium phosphate stand out as prominent calcium phosphates. This preference is rooted in a comprehensive understanding of their interactions with hydrogels and their behavior in diverse environmental conditions. In contrast, despite possessing commendable biological properties, the biomaterials of calcium phosphates like octacalcium phosphate (OCP), dicalcium phosphate dihydrate (DCPD) and dicalcium phosphate anhydrous (DCPA) present challenges in employing them as fillers for bioinks in bioprinting and tissue engineering. For example, dicalcium phosphates are stable at pH levels lower than 6, and with pH increases, dicalcium phosphate dihydrate transforms into octacalcium phosphate. This characteristic poses challenges in synthesizing dicalcium phosphate dihydrate/hydrogel materials for 3D bioprinting of tissue-engineering scaffolds. However, by utilizing an appropriate polymeric biomaterial, it is feasible to create a 3D-printed scaffold incorporating the dicalcium phosphate dihydrate phase, which will subsequently undergo conversion into octacalcium phosphate in vivo. While octacalcium phosphate holds promise for bone tissue engineering due to its ability to convert to hydroxyapatite through hydrolysis in physiological environments, the challenge in the control of its hydrolysis prompts the exploration of strategies involving its integration with hydrogels.

### 6.2. Recent Trends and Future Direction of CaP/Hydrogels for 3D Bioprinting

Even though hydroxyapatite, tricalcium phosphate and biphasic calcium phosphates are widely used calcium phosphates, some commercially manufactured biomaterials are based on these CaPs, they have certain limitations in their 3D-bioprinting applications. Because hydroxyapatite is not dissolved inside the body, hydroxyapatite-containing scaffolds are brittle, and additional supporting construction should be installed. Tricalcium phosphate is also not dissolved, but tricalcium-phosphate-based implants are resorbable through a cell-mediated process. However, achieving a balanced resorption rate relative to new bone formation poses other challenges. Notably, octacalcium phosphate, dicalcium phosphate dihydrate and dicalcium phosphate anhydrous have recently garnered attention due to their excellent osteoconductive and osteoinductive properties. Their potential to be synthesized through hydrogel polymerization and crosslinking processes is particularly noteworthy. Moreover, the transformative capability of OCP, DCPD and DCPA into hydroxyapatite holds promise for their application as integral components of bioinks for 3D bioprinting and biomimetics to the structures and compositions of the targeted tissues and organs. During hydrolysis, they undergo conversion into more stable hydroxyapatite, an insoluble inorganic component crucial for natural bone composition. Simultaneously, OCP, DCPD and DCPA promote osteoblast differentiation and cell conversion, enhancing connectivity between scaffolds and bone and facilitating accelerated bone formation and substitution. These phenomena indicate the importance of both 3D-bioprinting technology and modulation of bioink properties with the modulation of CaP/hydrogel species and their compositions in realizing the biomimetics of bone tissue regeneration.

## Figures and Tables

**Table 1:** Examples of recently investigated 3D-printed scaffolds incorporating HAp.

Polymer	Additive Used	HAp’s Concentration/Particle Size	Highlights	Scaffold Pore Size (Porosity, %)	Ref.
Alginate	Icariin	25% (*w*/*v*)/25–50 μm	Improved mechanical strength. Release of the icariin drug from the scaffold was slow and orderly sustained.	400–500 μm	[51]
GelMA	Ce-HAp	3% Ce-HAp (0.5%Ce mol.)/150–180 nm	Highly concentrated Ce-HAp (0.5 molar%)/GelMA composite shows the highest level of cell viability and proliferation potency along with a low cytotoxic potential.	126–138 µm for 20%GelMA-3%HC5 and 30%GelMA-3%HC5	[52]
GelMA	Mg/Zn-HAp	(Ca + Mg or Ca + Zn)/P of 1.67/ 70–140 nm (Mg) 50–120 nm (Zn)	Positive effect of magnesium and zinc on the osteogenic differentiation process.	100–200 µm (45% for 25%GelMA3%HAp-Zn and 53.6% for 30%GelMA-3%HAp-Zn)	[53]
Chitosan		70 wt%/nano size	Reinforces chitosan porous matrix.	160–275 μm	[54]
Poly (lactic-co-glycolic acid)	Polyvinyl alcohol	0%, 15%, 30%, 45%, and 60% (wt%)/n-HA sphere diameter: 3–10 μm	45 wt% HAp/PLGA had the highest compressive strength of more than 40 MPa, which was six times higher than that of the pure PLGA scaffold.	359.4 ± 12 μm	[55]
Nanocellulose-alginate			Graphene oxide (GO)-containing scaffolds have a higher swelling capacity compared to HAp-containing ones and promote a higher expression of osteogenic markers than HA. Hence, osteoinductive properties could be higher than HAp’s.	HAP 200–300 μm GO 400–500 μm	[56]
Gelatin	Graphene oxide (GO)	70% (*w*/*v*)/<200 nm	The 0.5% GO 3D-printed HA/gelatin scaffold shows increased compressive and flexural strength values by 15% and 22%, respectively. Additionally, GO’s reinforcer effects made the 3D-printed scaffolds compatible with cancellous bone.		[57]
Gelatin/collagen		<200 nm	Adjusting the duration of crosslinking allows for control of the stiffness of printed scaffolds.	430–550 μm	[58]
Chitosan/alginate		10% *w*/*v*/ <200 nm	The viability and attachment of the contracts on the scaffolds were enhanced using nHAp particles and alginate hydrogel. Chitosan-based scaffolds with nHAp particles had an improvement in elastic modulus and thermal stability behavior.	2–3 mm	[14]
Gelatin/hyaluronic acid		10% nHAp solution/particle size < 100 nm	High glucose can block the proliferation, migration and osteogenic differentiation of bone marrow stem cells (BMSCs). Both NG-EVs and HG-EVs can stimulate proliferation and migration, prevent apoptosis and promote osteogenic differentiation, but HG-EVs have a lesser effect than NG-EVs.	410–415 μm	[59]
Alginate-RGD	Sr-HA	0.5%, 1% and 2% (*w*/*v*) nHAp nanocrystals: - length 40–200 nm - width 10–60 nm SrHAp nanocrystals: - length 40–140 nm - width 10–50 nm	The ink can still maintain optimal extrusion and biocompatibility by adding a 1% *w*/*v* concentration, regardless of the type of particle.		[60]

**Table 3:** Summarizing table of CaP/hydrogel-based 3D-bioprinted scaffolds.

Calcium Phosphate	Hydrogel	Cells	Ref.
Hydroxyapatite	Collagen	Bone marrow mesenchymal stem cells (BMSCs)	[87]
Nanohydroxyapatite	Sodium alginate (SA)/gelatin (Gel)	Emodin-drug and mouse embryonic osteoblast precursor (MC3T3-E1) cells	[88]
Nanohydroxyapatite	Gelatin (Gel), quaternized chitosan (QCS)	Mesenchymal-stem-cell-derived exosomes	[89]
Hydroxyapatite	Collagen	MG63 cells and human adipose stem cells	[90]
α-TCP	Alginate	Mouse calvaria-derived preosteoblast cells (MC3T3)	[91]
Whitlockite and hydroxyapatite	Gelatin methacryloyl (GelMA) and alginate blended hydrogel (poly(ethylene glycol) as a hollowing agent in the first layer)	Human umbilical vein endothelial cells (HUVECs) and human mesenchymal stem cells (hMSCs)	[92]
Hydroxyapatite	Gelatin methacryloyl (GelMA), gelatin	MC3T3-E1 subclone 4 mouse calvaria osteoblast	[93]
Hydroxyapatite–magnetic iron oxide nanoparticles	Gelatin methacryloyl (GelMA)	Human PDLFs and human osteoblasts (hOBs)	[94]
α-TCP	Gelatin methacrylate (GelMA)	Mouse calvaria-derived preosteoblast cell line (MC3T3-E1)	[95]
Amorphous calcium phosphate micro/nanoparticles	Gelatin methacrylate (GelMA)	MC3T3, adipose-derived mesenchymal stem cells (AdMSC)	[96]
β-tricalcium phosphate	Polycaprolactone (PCL)	The patient’s autologous platelet-rich plasma (PRP)	[13]
β-tricalcium phosphate	Collagen	Human adipose stem cells (hASCs) and human umbilical vein endothelial cells (HUVECs)	[97]

## Data Availability

The original contributions presented in the study are included in the article, further inquiries can be directed to the corresponding author/s.
